# Supplementary material for: Geospatial analysis of regional variations in antibiotic consumption in Tanzania from 2020 to 2024 using the electronic logistics management information system
Source: Antimicrob Steward Healthc Epidemiol. 2026 Feb 16;6(1):e48. doi: 10.1017/ash.2025.10276 (PMC12912931; doi:10.1017/ash.2025.10276)
Supplement: Reuben et al. supplementary material [file S2732494X25102763sup001.docx]

**Supplementary Materials:**

**Geospatial Analysis of Regional Variations in Antibiotic Consumption in Tanzania from 2020 to 2024 Using the Electronic Logistics Management Information System**

William Reuben^1^, Florah Boniface Makenya^2^, Raphael Z. Sangeda^3*^, Daudi Ignasy Msasi^4^, Selesitine Ngoma^5^

^1^Department of Health, Social Development and Nutrition, President's Office, Regional Administration and Local Government, Tanzania

^2^School of Nursing and Public Health, University of Dodoma, Dodoma, Tanzania

^3^Department of Pharmaceutical Microbiology, Muhimbili University of Health and Allied Sciences, Tanzania

^4^Pharmaceutical Services Unit, Ministry of Health, Dodoma, Tanzania

^5^Department of Public Health and Community Nursing, University of Dodoma, Tanzania

Corresponding author: RZS^*^, sangeda@gmail.com

**Supplementary Tables**

**Supplementary Table 1.** Regional antibiotic distribution intensity (DDD per 1,000 inhabitants per day), Tanzania, 2020–2024.

| **Region** | **2020–2021** | **2021–2022** | **2022–2023** | **2023–2024** | **Region Total** | **% of Cumulative Total** | **% Change (2020–2021 to 2023–2024)** | **Cumulative %** |
| --- | --- | --- | --- | --- | --- | --- | --- | --- |
| Dar es Salaam | 15.37 | 8.25 | 4.98 | 9.36 | 37.95 | 5.8 | -39.1 | 5.8 |
| Ruvuma | 7.78 | 7.44 | 9.25 | 10.01 | 34.47 | 5.3 | 28.7 | 11.1 |
| Lindi | 4.38 | 11.00 | 9.58 | 9.04 | 34.00 | 5.2 | 106.7 | 16.3 |
| Iringa | 8.04 | 7.60 | 8.21 | 6.76 | 30.62 | 4.7 | -15.9 | 21.0 |
| Njombe | 7.60 | 8.13 | 6.68 | 7.33 | 29.74 | 4.6 | -3.5 | 25.6 |
| Mtwara | 4.71 | 9.39 | 7.37 | 7.75 | 29.22 | 4.5 | 64.7 | 30.1 |
| Dodoma | 6.70 | 7.01 | 7.80 | 7.51 | 29.02 | 4.5 | 12.1 | 34.6 |
| Tanga | 5.69 | 8.56 | 6.29 | 8.30 | 28.84 | 4.4 | 45.8 | 39.0 |
| Pwani | 7.64 | 7.18 | 6.69 | 7.26 | 28.77 | 4.4 | -4.9 | 43.4 |
| Kilimanjaro | 6.85 | 6.42 | 6.03 | 7.89 | 27.18 | 4.2 | 15.2 | 47.6 |
| Rukwa | 3.80 | 7.29 | 7.40 | 7.76 | 26.25 | 4.0 | 104.0 | 51.6 |
| Songwe | 5.65 | 6.24 | 7.46 | 6.56 | 25.92 | 4.0 | 16.1 | 55.6 |
| Kagera | 5.07 | 5.28 | 6.79 | 7.92 | 25.06 | 3.8 | 56.2 | 59.4 |
| Morogoro | 6.37 | 6.46 | 5.43 | 6.10 | 24.36 | 3.7 | -4.2 | 63.1 |
| Mbeya | 5.52 | 5.99 | 6.15 | 6.64 | 24.30 | 3.7 | 20.3 | 66.8 |
| Tabora | 3.95 | 5.73 | 6.28 | 6.69 | 22.65 | 3.5 | 69.3 | 70.3 |
| Mara | 3.69 | 4.64 | 6.64 | 7.20 | 22.17 | 3.4 | 95.4 | 73.7 |
| Manyara | 3.99 | 5.82 | 5.62 | 6.63 | 22.05 | 3.4 | 66.2 | 77.1 |
| Singida | 4.67 | 5.56 | 4.88 | 6.80 | 21.91 | 3.4 | 45.6 | 80.5 |
| Mwanza | 5.04 | 4.80 | 5.44 | 6.57 | 21.85 | 3.4 | 30.3 | 83.9 |
| Arusha | 3.97 | 5.64 | 4.85 | 6.35 | 20.81 | 3.2 | 60.2 | 87.1 |
| Kigoma | 4.31 | 4.65 | 5.23 | 6.37 | 20.57 | 3.2 | 47.6 | 90.3 |
| Shinyanga | 3.34 | 4.57 | 5.01 | 5.53 | 18.44 | 2.8 | 65.4 | 93.1 |
| Simiyu | 2.71 | 3.85 | 4.15 | 4.95 | 15.66 | 2.4 | 82.9 | 95.5 |
| Geita | 2.82 | 3.46 | 3.86 | 5.30 | 15.44 | 2.4 | 87.7 | 97.9 |
| Katavi | 2.73 | 2.81 | 3.93 | 4.21 | 13.68 | 2.1 | 54.4 | 100.0 |
| **National Total** | **142.37** | **163.76** | **162.01** | **182.79** | **650.92** | **100.0** | **--** | **--** |

*Note: Values show regional sums of defined daily doses (DDD) per 1,000 inhabitants per day (DID) based on eLMIS antibiotic supply data aggregated by fiscal year. The region total represents the cumulative DID across 2020–2024 and the percentage change reflects the relative change between the first and last fiscal years.*

**Supplementary Table 2.** Top antibiotics by consumption (DDD per 1,000 inhabitants per day), Tanzania, 2020–2024

| **Antibiotic (ATC code)** | **2020–2021** | **2021–2022** | **2022–2023** | **2023–2024** | **Cumulative Total** | **% of Cumulative Total** |
| --- | --- | --- | --- | --- | --- | --- |
| Amoxicillin (J01CA04) | 41.53 | 39.62 | 29.69 | 34.08 | 144.91 | 22.3 |
| Sulfamethoxazole + Trimethoprim (J01EE01) | 44.91 | 38.12 | 26.78 | 30.70 | 140.51 | 21.6 |
| Doxycycline (J01AA02) | 7.63 | 20.37 | 23.28 | 19.04 | 70.32 | 10.8 |
| Ciprofloxacin (J01MA02) | 12.56 | 17.58 | 20.16 | 19.78 | 70.08 | 10.8 |
| Erythromycin (J01FA01) | 10.86 | 14.97 | 15.07 | 14.28 | 55.17 | 8.5 |
| Metronidazole (J01XD01) | 6.48 | 10.38 | 16.66 | 20.48 | 53.99 | 8.3 |
| Ampicillin + Cloxacillin (J01CA51) | 6.92 | 7.67 | 11.58 | 16.11 | 42.27 | 6.5 |
| Azithromycin (J01FA10) | 1.05 | 3.39 | 6.67 | 12.08 | 23.19 | 3.6 |
| Amoxicillin + Clavulanic Acid (J01CR02) | 1.40 | 2.36 | 3.26 | 3.19 | 10.21 | 1.6 |
| Ceftriaxone (J01DD04) | 1.26 | 1.62 | 1.35 | 2.94 | 7.18 | 1.1 |
| Phenoxymethyl Penicillin (J01CE02) | 1.91 | 2.04 | 2.13 | 0.30 | 6.38 | 1.0 |
| Cephalexin (J01DB01) | 0.84 | 0.67 | 1.23 | 2.21 | 4.93 | 0.8 |
| Benzyl Penicillin (J01CE01) | 1.34 | 1.37 | 0.88 | 1.32 | 4.90 | 0.8 |
| Gentamicin (J01GB03) | 0.12 | 0.76 | 0.86 | 0.89 | 2.63 | 0.4 |
| Nitrofurantoin (J01XE01) | 0.20 | 0.42 | 0.54 | 1.33 | 2.49 | 0.4 |
| Ciprofloxacin + Tinidazole (J01RA11) | 1.48 | 0.11 | 0.01 | 0.00 | 1.60 | 0.2 |
| Tinidazole (J01XD02) | 0.22 | 0.13 | 0.05 | 1.13 | 1.53 | 0.2 |
| Cefixime (J01DD08) | 0.04 | 0.05 | 0.45 | 0.85 | 1.40 | 0.2 |
| Ampicillin (J01CA01) | 0.03 | 0.35 | 0.41 | 0.56 | 1.35 | 0.2 |
| Flucloxacillin + Amoxicillin (J01CF05) | 0.25 | 0.27 | 0.39 | 0.44 | 1.35 | 0.2 |
| **Subtotal (top 20)** | **144.64** | **173.09** | **175.99** | **187.52** | **681.24** | **≈ 99 % of total consumption** |

**Note:**
The following antibiotics were recorded to have negligible or no distribution during the study period (2020–2024): kanamycin (J01GB04), clindamycin (J01FF01), nalidixic acid (J01MB02), ceftazidime (J01DD02), tazobactam (J01CG02), **and amikacin (J01GB06).**

**Supplementary Table 3.** ATC Level 4 antibiotic classes--annual totals of defined daily doses per 1,000 inhabitants per day (DID) by fiscal year (2020–2024) and cumulative class totals.
Values derived from eLMIS facility-level supply data. The Class Total" rows represent the national sum per year; the rightmost column provides the cumulative DID per class across all years.

|  | **Fiscal Year** |  |  |  |  |
| --- | --- | --- | --- | --- | --- |
| **Level 4 Antibiotic (class)** | **2020-2021** | **2021-2022** | **2022-2023** | **2023-2024** | **Class Total** |
| Amphenicols (J01BA) | 0.009165 | 0.014371 | 0.013387 | 0.012936 | 0.049859 |
| Beta-lactamase inhibitors (J01CG) |  |  | 0 | 0 | 0 |
| Beta-lactamase resistant penicillins (J01CF) | 0.279362 | 0.269429 | 0.395662 | 0.440577 | 1.38503 |
| Beta-lactamase sensitive penicillins (J01CE) | 3.603444 | 3.581722 | 3.024911 | 1.618172 | 11.828249 |
| Carbapenems (J01DH) | 0.00058 | 0.00931 | 0.014997 | 0.015757 | 0.040644 |
| Combinations of antibacterials (J01RA) | 1.485495 | 0.123262 | 0.006455 | 0.002949 | 1.618161 |
| Combinations of penicillins, incl. beta-lactamase inhibitors (J01CR) | 1.403461 | 2.364825 | 3.255315 | 3.186898 | 10.210499 |
| Combinations of sulphonamides and trimethoprim, including derivatives (J01EE) | 44.909796 | 38.118795 | 26.783921 | 30.695668 | 140.50818 |
| First-generation cephalosporins (J01DB) | 0.838575 | 0.674016 | 1.227325 | 2.206148 | 4.946064 |
| Fluoroquinolones (J01MA) | 12.976689 | 17.957546 | 20.340317 | 19.861126 | 71.135678 |
| Fourth-generation cephalosporins (J01DE) | 0.088864 | 0.043165 | 0.055327 | 0.212251 | 0.399607 |
| Glycopeptide antibacterials (J01XA) | 0.000233 | 0.000713 | 0.00096 | 0.006894 | 0.0088 |
| Imidazole derivatives (J01XD) | 6.887814 | 11.194347 | 16.704434 | 21.605792 | 56.392387 |
| Lincosamides (J01FF) | 0.002429 | 0.000777 | 0.00135 | 0.000667 | 0.005223 |
| Macrolides (J01FA) | 11.995891 | 18.38516 | 21.948916 | 26.925127 | 79.255094 |
| Nitrofuran derivatives (J01XE) | 0.198007 | 0.418473 | 0.535158 | 1.333722 | 2.48536 |
| Other aminoglycosides (J01GB) | 0.120192 | 0.758478 | 0.86349 | 0.887055 | 2.629215 |
| Other antibacterials (J01XX) | 0.07758 | 0.157961 | 0.000084 | 0.048866 | 0.284491 |
| Other quinolones (J01MB) | 0 | 0 | 0 | 0 | 0 |
| Penicillins with extended spectrum (J01CA) | 48.484802 | 47.63442 | 41.66827 | 50.750147 | 188.537639 |
| Second-generation cephalosporins (J01DC) | 0.008357 | 0 | 0.004774 | 0.018324 | 0.031455 |
| Streptomycins (J01GA) | 0.000051 |  | 0.000201 |  | 0.000252 |
| Tetracyclines (J01AA) | 7.63499 | 20.368453 | 23.27892 | 19.035823 | 70.318186 |
| Third-generation cephalosporins (J01DD) | 1.361968 | 1.681346 | 1.886132 | 3.925111 | 8.854557 |
| **Class Total** | **142.367745** | **163.756569** | **162.010306** | **182.79001** | **650.92463** |

**Supplementary Table 4.** Forecast summary of national antibiotic consumption (defined daily doses per 1,000 inhabitants per day, DID), Tanzania, 2020–2027 (ARIMA and polynomial projections)

| **Fiscal Year** | **Observed DID** | **ARIMA Forecast** | **Polynomial Forecast** | **95% Confidence Interval (ARIMA)** |
| --- | --- | --- | --- | --- |
| 2020–2021 | 142.37 | -- | -- | -- |
| 2021–2022 | 163.76 | -- | -- | -- |
| 2022–2023 | 162.01 | -- | -- | -- |
| 2023–2024 | 182.79 | -- | -- | -- |
| 2024–2025 | -- | 193.4 | 195.6 | 188.1 – 198.7 |
| 2025–2026 | -- | 202.5 | 205.8 | 194.3 – 210.6 |
| 2026–2027 | -- | 211.1 | 215.2 | 200.4 – 222.8 |

**Note:** Forecasts derived from national annual DID values (2020–2024) using ARIMA (1,1,0) and second-degree polynomial models. Both models predicted continued growth in antibiotic consumption through 2027, with the polynomial model providing a marginally better fit (**R² = 0.97**).
